# Club Cell Heme Oxygenase-1 Deletion: Effects in Hyperoxia-Exposed Adult Mice

**DOI:** 10.1155/2020/2908271

**Published:** 2020-06-09

**Authors:** Katelyn Dunigan-Russell, Mary Silverberg, Vivian Y. Lin, Rui Li, Stephanie B. Wall, Qian Li, Teodora Nicola, John Gotham, David R. Crowe, Peter F. Vitiello, Anupam Agarwal, Trent E. Tipple

**Affiliations:** ^1^Division of Neonatology, Neonatal Redox Biology Laboratory, Department of Pediatrics, University of Alabama, USA; ^2^University of Alabama at Birmingham, Birmingham, AL, USA; ^3^Divison of Pathology, Department of Medicine, University of Alabama, USA; ^4^Department of Pediatrics, University of South Dakota Sanford School of Medicine, Sanford Research, Sioux Falls, ND, USA; ^5^Section of Neonatal-Perinatal Medicine, Department of Pediatrics, University of Oklahoma, Oklahoma City, OK, USA

## Abstract

Thioredoxin reductase-1 (TXNRD1) inhibition activates nuclear factor (erythroid-derived 2)-like 2 (Nrf2) responses and prevents acute lung injury (ALI). Heme oxygenase-1 (HO-1) induction following TXNRD1 inhibition is Nrf2-dependent in airway epithelial (club) cells *in vitro*. The influence of club cell HO-1 on lung development and lung injury responses is poorly understood. The present studies characterized the effects of hyperoxia on club cell-specific HO-1 knockout (KO) mice. These mice were generated by crossing *Hmox1* flox mice with transgenic mice expressing cre recombinase under control of the club cell-specific *Scgb1a1* promoter. Baseline analyses of lung architecture and function performed in age-matched adult wild-type and KO mice indicated an increased alveolar size and airway resistance in HO-1 KO mice. In subsequent experiments, adult wild-type and HO-1 KO mice were either continuously exposed to >95% hyperoxia or room air for 72 h or exposed to >95 hyperoxia for 48 h followed by recovery in room air for 48 h. Injury was quantitatively assessed by calculating right lung/body weight ratios (g/kg). Analyses indicated an independent effect of hyperoxia but not genotype on right lung/body weight ratios in both wild-type and HO-1 KO mice. The magnitude of increases in right lung/body weight ratios was similar in mice of both genotypes. In the recovery model, an independent effect of hyperoxia but not genotype was also detected. In contrast to the continuous exposure model, right lung/body weight ratio mice were significantly elevated in HO-1 KO but not wild-type mice. Though club cell HO-1 does not alter hyperoxic sensitivity in adult mice, it significantly influences lung development and resolution of lung injury following acute hyperoxic exposure.

## 1. Introduction

Thioredoxin reductase-1 (TXNRD1) inhibition activates nuclear factor (erythroid-derived 2)-like 2 (Nrf2) responses [1]. Pharmacologic TXNRD1 inhibition with aurothioglucose (ATG) improves alveolarization in hyperoxia-exposed neonatal mice [2]. ATG consistently attenuates lung injury and improves survival in adult murine models [3, 4].

Hyperoxic exposure leads to increased production of reactive oxygen species (ROS), that in turn, damages lipid barriers, proteins, and nucleic acids [5]. In the setting of persistent ROS production, antioxidant defenses become depleted [2]. This leads to irreversible lung injury characterized by loss of epithelial barrier integrity, inflammatory cell infiltration, leakage of proteinaceous fluid into the alveolar space, and the appearance of hyaline membranes [6, 7]. Therapeutic oxygen treatment is associated with a myriad of negative effects including depressed respiratory drive, decreased gas exchange efficiency, and alveolar collapse [6].

HO-1 catalyzes the rate-limiting step in the reduction of free heme to form carbon monoxide, ferrous iron, and biliverdin [8, 9]. HO-1 can be either protective or detrimental depending upon the pathology or organ system [10–14]. In the lung, HO-1 is necessary for proper development as demonstrated by alveolar simplification in mice lacking HO-1 expression [15]. In lung macrophages, HO-1 is essential for clearance of pathogens and cellular debris [16]. HO-1 is induced by a variety of stimuli including heavy metals, cytokines, and lipopolysaccharide (LPS) [17, 18].

Nonciliated club cells make up ~9% of the total population of airway epithelial cells in the human lung [19, 20]. Club cell secretory protein (CCSP, CC10, Scgb1a1) is a 10 kd protein member of the secretoglobin family [19]. Scgb1a1 has phospholipase A2 inhibitory and immunomodulatory functions [20]. During periods of damage and repair, club cells act as progenitors for type 2 pneumocytes that can, in turn, differentiate into type 1 pneumocytes [20, 21]. In neonatal murine, adult murine, and human lungs, TXNRD1 is most abundantly expressed in club cells and macrophages [4].

Our previous work in vitro in immortalized murine club cells revealed that HO-1 is disproportionately induced by TXNRD1 inhibition, when compared to other Nrf2-regulated genes [22]. Thus, we speculate that the protective effects of TXNRD1 inhibition are mediated, at least in part, by HO-1 [22]. The present studies were designed to evaluate the contribution of club cell HO-1 to hyperoxic injury and repair in adult mice.

## 2. Materials and Methods

### 2.1. Generation of Transgenic Mice

Animal protocols were approved by the Institutional Animal Care and Use Committee at the University of Alabama at Birmingham (UAB). Mice were handled in accordance with the National Institutes of Health guidelines. C57Bl/6 ROSA^mT/mG^ (B6.129 (Cg)-Gt(ROSA)26S^tm4(ACTB-tdTomato,-EGFP)Lou^/J, Jackson Laboratory, Stock No. 007676) mice were crossbred with *Scgb1a1*-Cre mice (T. Mariani, U. of Rochester) to generate *Scgb1a1*^EGFP^ mice [23–25]. *Scgb1a1*-Cre mice were also crossed with *Hmox1*^fl/fl^ mice (A. Agarwal, UAB) to generate club cell-specific HO-1 knockout (KO) mice (*Hmox1*^*∆*/*∆*^). All mice were on a C57Bl/6J background. Inbred generations of these crosses were bred to create a stable mouse line of each knockout mouse (Table 1).

### 2.2. Animals

Mice were euthanized using a single intraperitoneal injection of ketamine/xylazine (150/15 mg/kg). Wild-type and Scgb1a1-EGFP lungs from 3, 7, 10, and 14-day old mice were inflated with optimal cutting temperature (OCT) compound at 25 cm H_2_O and allowed to equilibrate for 15 minutes. Scgb1a1-*Hmox1^f/f^* (HO-1 WT) and Scgb1a1-*Hmox1^Δ/Δ^* (HO-1 KO) mice were harvested at 7-12 weeks of life for analyses of lung function (total lung resistance and static lung compliance) using a flexiVent apparatus (SCIREQ, Montreal, QC, Canada) [26–28]. Hyperoxic exposures were performed in an A-Chamber with oxygen levels regulated by a ProOx 360 controller (BioSpherix, Parish, NY).

### 2.3. Anti-GFP and H&E Staining of Lung Tissue

Tissues were processed on a Leica 300 ASP tissue processor (Buffalo Grove IL) and sectioned at 5 *μ*m. The BenchMark XT automated slide staining system (Medical Systems Inc., Tucson, AZ) was used for antibody labeling with chicken anti-GFP (1 : 250, ab13970, Abcam, Cambridge UK). Antigen retrieval was performed using the Ventana CC1 solution (950-124, Roche, Basel, Switzerland), a basic pH Tris-based buffer, and the Ventana iView DAB detection kit (760-091, Roche) was used as the chromogen. Slides were counterstained with hematoxylin and scanned using a Leica Aperio VERSA digital slide scanner.

### 2.4. Morphometric and Injury Assessments

Mice were weighed at euthanasia, the right main stem bronchus was ligated, and right lungs removed and weighed. The trachea was cannulated, and left lungs were inflation fixed with 10% buffered formalin at a pressure of 25 cm H_2_O for 15 min. After equilibration, lungs were removed and fixed in buffered formalin overnight. Morphometric analyses for mean linear intercept (MLI) and radial alveolar count (RAC) were performed [29–32]. Assessments of H&E-stained lung sections were blindly performed by a pathologist, and indices of injury were scored using American Thoracic Society guidelines [33].

### 2.5. Statistics

Data were analyzed using GraphPad Prism 8.0 (La Jolla, CA). All data (expressed as mean ± SD) were tested for homogeneity of variances and were log-transformed where indicated. Parametric data were analyzed by unpaired *t*-test or analysis of variance (ANOVA) followed by Tukey's multiple-comparison post hoc test. Statistical significance was accepted at *p* < 0.05.

## 3. Results

### 3.1. Cre-Mediated Recombination in *Scgb1a1-*Cre Mice

**Figure 1:**
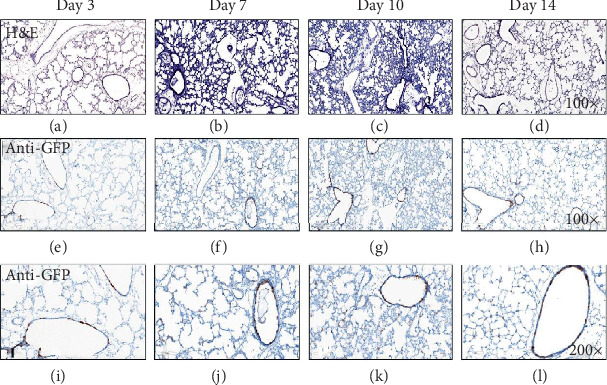
Timeline of *Scgb1a1*^Cre^-mediated recombination in newborn lungs. Representative lung sections from 3, 7, 10, and 14 d *Scb1a1*-EGFP mice were immunostained as described in Methods. EGFP positivity is indicated by brown staining (*n* = 3).

Though we attempted to characterize HO-1 expression in lung sections from *Scgb1a1*-*Hmox1*^*∆*/*∆*^ mice, the intensity of HO-1 expression in macrophages impaired our ability to titrate our antibodies to levels appropriate for detection of immunostained epithelia. Timing and specificity of *Scgb1a1*-Cre-mediated recombination for the mice used in the present studies has been reported previously [23–25]. Nevertheless, for the purpose of scientific rigor, we validated the localization and timing of cre-mediated recombination events by breeding *Scgb1*-EGFP reporter mice. Representative H&E and anti-GFP images of lung sections from days 3, 7, 10, and 14 days of life are shown in Figure 1. Recombination was qualitatively assessed by identifying localization of anti-GFP antibody staining location over time. GFP positivity was detected in the bronchiolar epithelium at days 3 (Figures 1(e) and 1(i)) and 7 (Figures 1(f) and 1(j)). By day 10, GFP-positive immunostaining was also detectable in bronchiolar and alveolar cells (Figures 1(g) and 1(k)). Similar staining patterns were also present at day 14 (Figures 1(h) and 1(l)). Consistent with prior reports, our data confirmed that recombination events in this specific line of *Scgb1a1*-Cre mice are complete by the end of alveolarization [23].

### 3.2. Club Cell HO-1 Deletion Causes Alterations in Lung Development

**Figure 2:**
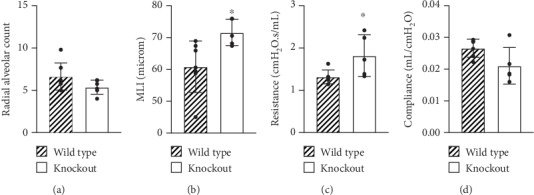
Effects of club cell HO-1 deletion on lung architecture and function in adult mice. (a) Radial alveolar count (RAC) and (b) mean linear intercept (MLI) were determined in H&E-stained lungs from adult HO-1 KO and wild-type mice. Data (mean ± SD, *n* = 6 − 7) were analyzed by unpaired *t*-test (∗*p* = 0.0221 vs wild-type). (c) Resistance and (d) compliance were assessed using flexiVent. Data (mean ± SD, *n* = 5 − 6) were analyzed by unpaired *t*-test (∗*p* = 0.0419 vs wild-type).

Lung sections from age-matched adult HO-1 KO mice and wild-type controls were evaluated to define baseline effects of club cell HO-1 deletion. Radial alveolar counts (RAC) and mean linear intercepts (MLI) were determined to quantitatively evaluate lung architecture. RAC was not different between wild-type (6.6 ± 1.6) and HO-1 KO mice (5.4 ± 0.8; Figure 2(a)). In contrast, our analyses revealed that MLI was significantly greater in HO-1 KO mice (71.6 ± 4.1 *μ*M) than in wild-type controls (60.9 ± 8.1 *μ*M; Figure 2(b)).

Analyses were also performed to formally assess lung function. Our data indicated that resistance was significantly greater in HO-1 KO mice (1.8 ± 0.5 cm H_2_O·s/mL) when compared to age-matched wild-type controls (1.3 ± 0.2 cm H_2_O · s/mL; Figure 2(c)). In contrast, airway compliance was not different between HO-1 KO (0.021 ± 0.006 mL/cm H_2_O) and wild-type controls (0.027 ± 0.003 mL/cm H_2_O; Figure 2(d)).

### 3.3. Hyperoxic Exposure Causes Lung Injury in HO-1 KO and Wild-Type C57Bl/6J Mice

**Figure 3:**
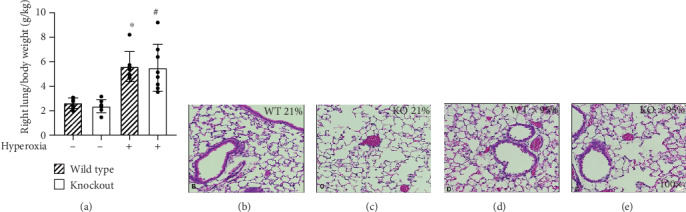
Hyperoxia causes lung injury in HO-1 KO and wild-type mice. Adult (7-12 wks) wild-type and HO-1 KO mice were continuously exposed to >95% O_2_ for 72 h. (a) Right lung/body weight ratios were calculated. Data (mean ± SD, *n* = 7 − 8) were analyzed by two-way ANOVA followed by Tukey's post hoc analysis (∗*p* = 0.0007 vs wild-type/room air; ^#^*p* = 0.0003 vs HO-1 KO/room air). An independent effect of hyperoxia was detected (*p* < 0.0001). (b–e) Representative H&E stained sections.

**Figure 4:**
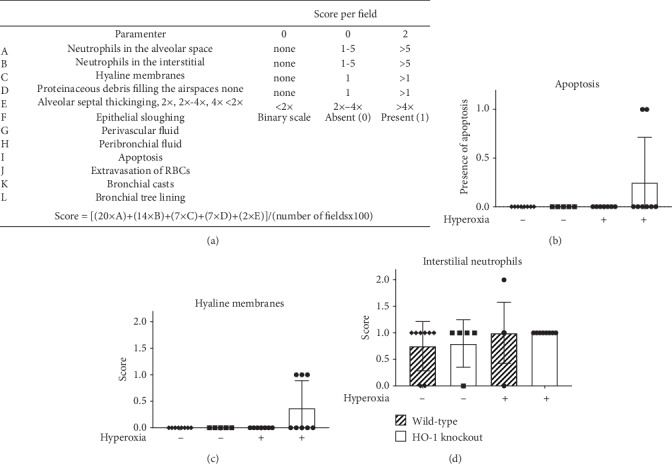
Lung scoring of >95% oxygen for 72 h in HO-1 KO and wild-type mice. Adult (7-12 wks) wild-type and HO-1 KO mice were exposed to continuous >95% oxygen for 72 h as described in the Methods. Lung scoring was blindly performed by a certified pathologist and scored using the American Thoracic Society Standard Lung Scoring for Acute Lung Injury.

Wild-type and HO-1 KO mice were exposed to room air or >95% O_2_ for 72 h. As previously published by our group, right lung/body weight ratios were calculated as an index of pulmonary injury [2, 4, 34]. There was an effect of hyperoxia but no effect of strain on right lung/body weight ratios in hyperoxia-exposed HO-1 KO and wild-type mice. Hyperoxic exposure caused a significant increase in right lung/body weight ratios in wild-type mice (5.6 ± 1.2 vs. 2.6 ± 0.4 g/kg; Figure 3(a)). Also in HO-1 KO mice, right lung/body weight ratios were significantly greater following hyperoxia when compared to room air controls (5.5 ± 1.9 vs. 2.4 ± 0.5 g/kg; Figure 3(a)). H&E-stained slides from wild-type and HO-1 KO mice exposed to >95% O_2_ for 72 h (Figure 3(b)) were also evaluated by a certified pathologist blinded to treatment groups using guidelines established by the American Thoracic Society (Figure 4(a)) [33]. We did not detect significant effects of strain or hyperoxia on apoptosis, hyaline membranes, or interstitial neutrophils. There was, however, evidence of apoptosis and hyaline membranes in hyperoxia-exposed HO-1 KO mice only (Figures 4(b) and 4(c)).

### 3.4. Deletion of Club Cell HO-1 Does Not Alter Recovery in Hyperoxia-Exposed Mice

**Figure 5:**
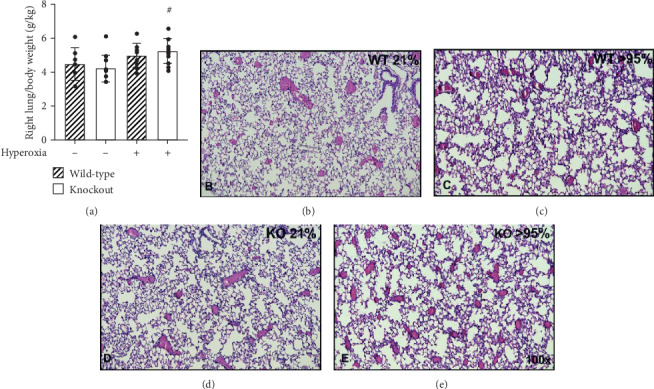
Effects of 48 h recovery from hyperoxic exposure in HO-1 KO and wild-type mice. Adult (7-12 wks) wild-type and HO-1 KO mice were continuously exposed to >95% O_2_ for 48 h followed by a return to room air for 48 h. Control mice remained in room air for 48 h. (a) Right lung/body weight ratios were calculated. Data (mean ± SD, *n* = 7 − 14) were analyzed by two-way ANOVA followed by Tukey's post hoc analysis (^#^*p* = 0.0204 vs HO-1 KO/room air). An independent effect of hyperoxia was detected (*p* < 0.0054). (b–e) Representative H&E stained sections.

Club cells act as progenitor cells following lung damage [15, 20]. Thus, HO-1 KO and wild-type mice were exposed to >95% oxygen for 48 h, followed by recovery in room air for 48 h. We detected an independent effect of hyperoxia but no effect of strain on right lung/body weight ratios (Figure 5). In wild-type mice, right lung/body weight ratios were not different between room air and hyperoxia/recovery groups (4.5 ± 1.0 vs. 5.0 ± 0.7 g/kg). In contrast, right lung/body weight ratios were significantly greater in hyperoxia/recovery HO-1 KO mice when compared to room air HO-1 KO controls (5.3 ± 0.7 vs. 4.2 ± 0.8 g/kg) (Figure 5).

## 4. Discussion

The present studies were designed to define the contribution of club cell HO-1 on pulmonary responses to hyperoxia in adult mice. We first confirmed that the *Scgb1a1-*Cre mouse elicits recombination in airway and alveolar epithelia by 14 d. We then used these mice to generate HO-1 KO mice (Table 1) and found baseline differences in lung architecture and airway resistance when compared to wild-type controls. In adult mice exposed continuously to >95% O_2_ for 72 h, right lung/body weight ratios were increased by hyperoxia to similar degrees in both HO-1 KO and wild-type control mice. In mice exposed to >95% O_2_ for 48 h, followed by room air recovery for 48 hours, right lung/body weight ratios were elevated in HO-1 KO but not wild-type control mice. Though club cell HO-1 does not alter hyperoxic sensitivity in adult mice, club cell HO-1 specifically influences lung development and resolution of lung injury following acute hyperoxic exposure.

We verified that cre-mediated recombination events occur during the course of alveolar development (Figures 1(d), 1(h), and 1(l)). We detected baseline increases in MLI, but not RAC in HO-1 KO mice when compared to wild-type mice (Figure 2(b)) implicating epithelial HO-1 as a regulator of epithelial differentiation and alveolarization. These data are in contrast to findings in mice bearing a germline deletion in *Hmox1* in which global HO-1 deficiency was associated with decreased RAC, though MLI was not assessed in the global HO-1 KO mice [35]. We searched the Lung Gene Expression in Single-cell (LungGENS) database to better understand *Hmox1* expression in the developing lung [36]. At postnatal day 1, *Hmox1* is primarily expressed in lung epithelial precursors, including club cells. By postnatal day 3, and thereafter, *Hmox1* expression is tightly restricted to myeloid cells. These findings are consistent with our immunohistochemical data (not shown) in which the degree of macrophage HO-1 expression was too robust to permit evaluation of epithelial HO-1 expression in our HO-1 KO mice. Lung macrophages influence alveolar development [37, 38]. Our data support a role for epithelial HO-1 expression in normal lung morphogenesis. We speculate that differences between global- and epithelial-specific HO-1 deletions are likely to be driven by the timing and specificity of cre-mediated recombination in our transgenic HO-1 KO mice and/or the influence of myeloid cell HO-1 expression.

In addition to baseline differences in alveolarization, club cell HO-1 deletion also impacted lung function. Though epithelial HO-1 deletion increased airway resistance in adult mice when compared to wild-type mice (Figure 2(c)), static lung compliance was not different between the groups (Figure 2(d)). These data most likely represent cross-talk between HO-1-deficient epithelia and other cell types during airway differentiation. The absence of an effect on static lung compliance suggests that these effects are likely limited to airway but not total lung function.

Hyperoxic exposure causes consistent lung injury in adult mice. We have previously shown that HO-1 expression is substantially increased in the lungs of newborn and adult mice exposed to hyperoxia following TXNRD1 inhibition with ATG. TXNRD1 is most abundantly expressed in club cells and macrophages [39, 40]. To understand the fundamental contribution of club cell HO-1 expression, we exposed wild-type and HO-1 KO mice to >95% O_2_ for 72 h and assessed lung injury by calculating right lung/body weight ratios. An independent effect of hyperoxia but not genotype was detected (Figure 3). Lung injury scoring at a single time point was utilized to evaluate the impact of hyperoxic exposure. We do not interpret the lack of an effect of hyperoxia on apoptosis, hyaline membranes, or interstitial neutrophil counts as a lack of acute lung injury. In our experience, C57Bl/6 mice begin to exhibit mortality by 96 h of continuous exposure to >95% O_2_. Because we did not know the hyperoxic sensitivity of our HO-1 KO mice, we chose an evaluation timepoint that was earlier than 96 h. Though there was no effect of genotype, evidence of apoptosis (Figure 4(b)) and hyaline membranes (Figure 4(c)) were only observed in hyperoxia-exposed HO-1 KO mice. Thus, the lack of differences is most reasonably attributable to long-term adaptation to HO-1 deficiency in HO-1 KO mice, sensitivity of the methods used, and/or our choice of 72 h as a time for analyses. In addition, macrophage-dependent responses significantly contribute to hyperoxic responses in the adult lung. As in lung development, we speculate that the influence of macrophage HO-1 may be more significant than epithelial HO-1 expression.

Given the role of club cells in repair processes from lung injury, we also exposed HO-1 KO and wild-type mice to >95% O_2_ for 48 h and allowed them to recover in room air for 48 h. Two-way ANOVA indicated a significant effect of hyperoxia on lung injury as assessed by right lung/body weight ratios (Figure 5(a)). In contrast to the 72 h hyperoxic exposure, we detected a significant increase in right lung/body weight ratios in HO-1 KO but not wild-type mice in the hyperoxia/recovery (Figure 5(a)). Thus, our findings indicate that club cell HO-1 contributes to the resolution of lung injury following acute hyperoxic exposure.

In conclusion, club cell HO-1 contributes to alveolar and airway development in mice. Though acute responses to hyperoxic exposure do not appear to be influenced by HO-1, club cell HO-1 appears to be necessary for resolution of acute lung injury induced by short-term hyperoxic exposure. Our data also suggest that the influence of myeloid/macrophage HO-1 expression is likely to be proportionately greater than epithelial HO-1. Future studies will utilize macrophage-specific HO-1 deletion to test this hypothesis. Nonetheless, our data suggest that therapies that enhance pulmonary HO-1 expression, such as TXNRD1 inhibition, may improve outcomes in patients with acute lung injury.

## Figures and Tables

**Table 1:** Crossing strategies and resultant genotypes of transgenic mice used in the present studies.

Cross	Genotype	Title
Scgb1a1-Cre x Rosa^mT/mG^	Scgb1a1-mT	Wild type
	Scgb1a1-EGFP	EGFP+
Scgb1a1-Cre x Hmox1^f/f^	Scgb1a1-Hmox1^f/f^	Wild type
	Scgb1a1-Hmox1^*Δ*/*Δ*^	HO-1 knockout

## Data Availability

No publicly available data were used for this manuscript.

## Abbreviations

HO-1: Heme oxygenase 1 protein
*Hmox1*: Heme oxygenase 1 gene
RAC: Radial alveolar count
MLI: Mean linear intercept
CC10, Scgb1a1: Club cell protein
Nrf2: Nuclear factor (erythroid-derived 2)-like 2
LPS: Lipopolysaccharide
ROS: Reactive oxygen species
TXNRD1: Thioredoxin reductase-1
ATG: Aurothioglucose
NQO1: NADPH quinone oxidoreductase-1.
